# Supplementary material for: Prethermalization in one-dimensional quantum many-body systems with confinement
Source: Nat Commun. 2022 Dec 10;13:7663. doi: 10.1038/s41467-022-35301-6 (PMC9741589; doi:10.1038/s41467-022-35301-6)
Supplement: Supplementary file 1 — Supplementary Information [file 41467_2022_35301_MOESM1_ESM.pdf]

Supplementary Information
Prethermalization in one-dimensional quantum many-body systems with confinement

Stefan Birnkammer, Alvise Bastianello, Michael Knap

1. The weakly confined transverse Ising chain 1

2. Thermodynamics for mesonic systems 3

3. Initializing moving mesons by modulated pulses of the transverse field 6

4. Lattice gauge theories: the example of the U(1) quantum link model 7

5. Exact diagonalization in the few kinks subspace 9
 1. Benchmarking exact diagonalization in the few kinks subspace with tensor networks 11

References 12

1. THE WEAKLY CONFINED TRANSVERSE ISING CHAIN

For the sake of completeness, in this section we summarize the basics of the Ising spin chain in a weak longitudinal field, discussing the dynamics projected in the subspace with a fixed number of fermions and the quench dynamics in the protocol of interest.

The Ising chain in pure transverse field — As a starting point, we consider first the Ising chain in a pure transverse field ($h_\parallel = 0$ in Eq. (1)). This is a well-known exactly solvable model equivalent to free fermions, see e.g. Ref. [1] for a extensive discussion. Spinless fermions $\{\hat{c}_i, \hat{c}_j^\dagger\} = \delta_{i,j}$ are defined through a Jordan-Wigner transformation

$$\frac{\hat{\sigma}_j^x + i\hat{\sigma}_j^y}{2} = \exp\Big(i\pi \sum_{i<j} \hat{c}_i^\dagger \hat{c}_i\Big)\hat{c}_j^\dagger\,. \tag{1}$$

The Ising Hamiltonian commutes with the parity operator $\hat{P} = \prod_j \hat{\sigma}_j^x$, thus the Hilbert space splits into two disconnected parts of different parity $\hat{P} = \pm 1$: the spinless fermions obey different boundary conditions depending on the parity sector, but this subtlety can be ignored in the infinite volume limit. The transverse Ising Hamiltonian is then diagonalized in the Fourier basis after a Bogoliubov rotation

$$\begin{pmatrix} \hat{c}_j \\ \hat{c}_j^\dagger \end{pmatrix} = \int \frac{\mathrm{d}k}{\sqrt{2\pi}} e^{ikj} \begin{pmatrix} \hat{\alpha}(k) \\ \hat{\alpha}^\dagger(-k) \end{pmatrix} = \int \frac{\mathrm{d}k}{\sqrt{2\pi}} e^{ikj} U_{\theta_k} \begin{pmatrix} \hat{\gamma}(k) \\ \hat{\gamma}^\dagger(-k) \end{pmatrix}, \qquad U_{\theta_k} = \begin{pmatrix} \cos\theta_k & i\sin\theta_k \\ i\sin\theta_k & \cos\theta_k \end{pmatrix}, \tag{2}$$

where the modes $\hat{\gamma}(k)$ obey the canonical anticommutation rules $\{\hat{\gamma}(k), \hat{\gamma}^\dagger(q)\} = \delta(k-q)$. The angle θ_k parametrizes the Bogoliubov rotation and the choice

$$\theta_k = -\frac{1}{2i}\log\left(\frac{h_\perp - e^{ik}}{(\cos k - h_\perp)^2 + \sin^2 k}\right) \tag{3}$$

diagonalizes the Hamiltonian $\hat{H} = \int \mathrm{d}k\, \epsilon(k)\hat{\gamma}^\dagger(k)\hat{\gamma}(k) + \text{const.}$ with $\epsilon(k) = 2\sqrt{(\cos k - h_\perp)^2 + \sin^2 k}$. In the fermion language, the Hilbert space can be described as a Fock space built by acting with the creation operators on the vacuum $|0\rangle$ (defined as $\hat{\gamma}(k)|0\rangle = 0$), which is also identified with the ground state of the chain. Homogeneous quantum quenches in the transverse Ising has been first addressed in Ref. [1]: in this framework, one initializes the system in the ground state for a certain transverse field $\tilde{h}_\perp$ and brings the system out of equilibrium by changing $\tilde{h}_\perp \to h_\perp$. By using that the initial state is annihilated by the modes that

diagonalize the prequench Hamiltonian and that the pre- and post-quench modes are connected through a Bogoliubov rotation, one can write the initial state in a simple squeezed form

$$|\psi\rangle \propto \exp\left[\int_0^{\pi} \mathrm{d}k\, K(k)\hat{\gamma}^\dagger(k)\hat{\gamma}^\dagger(-k)\right]|0\rangle\,, \tag{4}$$

with the wavefunction $K(k)$ determined by the difference of the pre and post quench Bogoliubov angles $K(k) = -i\tan(\theta_k^{\text{post}} - \theta_k^{\text{pre}})$. By similar means, in Section 3 we will determine the initial state obtained after a staggered pulse in the transverse magnetization.

Effects of weak confinement: the projected dynamics — We now consider the activation of a non-trivial longitudinal field $h_\parallel$. Following Ref. [2], one expresses the full Hamiltonian in the basis of the modes of the transverse part. Hence, let $|\{k_i\}_{i=1}^N\rangle$ be a multi-fermionic state: apart from a non-essential constant offset, the Hamiltonian reads

$$\hat{H}|\{k_i\}_{i=1}^N\rangle = \left(\sum_{i=1}^N \epsilon(k_i)\right)|\{k_i\}_{i=1}^N\rangle + h_\parallel \sum_{M=1}^{\infty}\frac{1}{M!}\int_{-\pi}^{\pi}\frac{\mathrm{d}^M q}{(2\pi)^M}\,2\pi\delta\left(\sum_{j=1}^M q_j - \sum_{i=1}^N k_i\right)|\{q_j\}_{j=1}^M\rangle\langle\{q_j\}_{j=1}^M|\hat{\sigma}_0^z|\{k_i\}_{i=1}^N\rangle\,. \tag{5}$$

The matrix elements of the longitudinal magnetization $\langle\{q_j\}_{j=1}^M|\hat{\sigma}_0^z|\{k_i\}_{i=1}^N\rangle$ —also called form factors— are known and, by means of a repeated use of the Wick theorem, are entirely encoded in the two-fermion form factors $\langle k_1k_2|\hat{\sigma}_0^z|0\rangle$, $\langle 0|\hat{\sigma}_0^z|k_1,k_2\rangle$ and $\langle k_1|\sigma_0^z|k_2\rangle$, see Ref. [2] for further details. The Hamiltonian in Supplementary Eq. (5) is valid for arbitrary values of the longitudinal field $h_\parallel$. However, in the limit of weak confinement $|h_\parallel| \ll \min_k \epsilon(k)$, important simplifications can be invoked. As we mentioned in the main text, the effective dynamics conserves the number of fermions on very large time scales, hence we can project the dynamics of Supplementary Eq. (5) in the number-conserving space. Secondly, the interaction in the number-conserving sector splits into a long-range linear potential (inducing the confinement) plus short range corrections [2] (see also Ref. [3]). Short range corrections are negligible when compared with the linear term (and are furthermore linearly suppressed for $h_\perp$ small). With these considerations, in the two particle sector one reaches the simple Hamiltonian

$$\hat{H}_{\text{2pt}} = \int \mathrm{d}k_1\mathrm{d}k_2[\epsilon(k_1)+\epsilon(k_2)]|k_1,k_2\rangle\langle k_1,k_2| + \sum_{j_1,j_2} 2h_\parallel\bar{\sigma}|j_1-j_2||j_1,j_2\rangle\langle j_1j_2|\,, \tag{6}$$

where the potential term is more conveniently expressed in the real basis $|j_1,j_2\rangle \equiv \int \frac{\mathrm{d}k_1\mathrm{d}k_2}{2\pi}e^{ikj_1+ik_2j_2}|k_1,k_2\rangle$. The expression can be directly generalized beyond the two fermions sector. We notice that for small transverse fields, the kinetic energy reads $\epsilon(k) \simeq 2 - 2h_\perp\cos(k) + \dots$ and the kinetic term becomes a simple nearest-neighbor hopping. For the sake of simplicity, in Fig. 2 of the main text we consider this limit, where in addition fermions can be identified with sharp domain walls in the Ising chain. The more general case of finite $h_\perp$ can be also addressed in a similar way. When considering the quench protocol of interest, the state of Supplementary Eq. (4) can be used as the initial condition of the dynamics: this proposal has been originally made in Ref. [4]. However, while at late time the Schwinger mechanism is extremely suppressed, it has been recently understood that the activation of a longitudinal field non-trivially affects the number of fermions [3]. This additional production of excitations ceases after a short time transient and effectively renormalizes the wavefunction

$$K(k) \to \mathcal{K}(k) = -i\tan(\theta_k^{\text{post}} - \theta_k^{\text{pre}}) - ih_\parallel\bar{\sigma}v(k)/\epsilon^2(k)\,, \tag{7}$$

with $v(k) = \partial_k\epsilon(k)$ being the group velocity. While this renormalization can be important to check meson conservation for relatively strong longitudinal field (eg Figs. 1(c)), its effect is negligible for the parameter choice of Figs. 3 and 4 and it is thus neglected.

Quantization of the mesonic states — Mesons have internal degrees of freedom associated with the different energy levels of the two-particle problem described by $\hat{H}_{\text{2pt}}$ Supplementary Eq. (6). In the limit of weak transverse field $\epsilon(k) \simeq 2 - 2h_\perp\cos(k) + \mathcal{O}(h_\perp^2)$ the eigenfunctions and energies can be exactly computed by means of Bessel functions [2]. For finite transverse field, a simple analytical solution is not available, but $\hat{H}_{\text{2pt}}$ can be easily numerically diagonalized (see eg. Ref. [3]). Nonetheless, the limit where the longitudinal field is much weaker than the transverse one $h_\parallel \ll h_\perp$ is amenable of semiclassical methods. Already in the seminal paper [4], the semiclassical quantization of meson energies has been observed to be in very good agreement with numerical data, even far from the extreme limit $h_\parallel \ll h_\perp$. Therefore, further motivated by our goal to describe the prethermal phase observed in the classical regime, we briefly review the semiclassical quantization of mesonic masses [2]: the pure classical limit is then recovered in the limit of vanishing longitudinal field, where the quantized energies merge in a continuum.

In order to find the momentum-dependent energy levels $\{\mathcal{E}(J,k)\}_J$, let us address the classical two-fermion problem associated with Supplementary Eq. (6). It is convenient to consider the center-of-mass and the relative coordinates, (X,k) and (x,q) respectively. For this choice of coordinates the 2−particle classical Hamiltonian takes the simple form

$$H_{\text{2pt}}(k,q,X,x) = \epsilon(k/2+q) + \epsilon(k/2-q) + \chi|x| \equiv \omega(q,k) + \chi|x| \tag{8}$$

where we introduced the notation $\chi = 2h_{\parallel}\bar{\sigma}$ and $\omega(k,q) = \epsilon(k/2+q) + \epsilon(k/2-q)$. The total momentum k is conserved.

Generally $q(t)$ and $x(t)$ describe an oscillating motion associated to the breathing of the fermion bound state. The latter is formally captured by

$$q(t) = \begin{cases} q_a(E,k) - \chi t & t \in [0, t_1] \\ q_b(E,k) + \chi(t - t_1) & t \in [t_1, 2t_1] \end{cases} \qquad x(t) = \begin{cases} \chi^{-1}\big(E - \omega(k, q(t))\big) & t \in [0, t_1] \\ -\chi^{-1}\big(E - \omega(k, q(t))\big) & t \in [t_1, 2t_1] \end{cases} \tag{9}$$

Here we introduced the points (q_a, q_b) as the turning points of the classical problem, where the total energy of the relative motion is stored in kinetic energy, i.e. the relative distance of the fermions vanishes. These turning points are reached after multiples of the time period $t_1 = (q_a - q_b)/\chi$. Depending on the choice for k the function $\omega(k,q)$ will either resemble the shape of a single or double-well potential in q. This entails two fundamentally different cases for the turning points q_a, q_b. For small values of k, $\omega(k,q)$ looks like a single well potential symmetric in $q \to -q$. The turning points will consequentially share this reflection symmetry around $q = 0$ and satisfy the constraint $q_b = -q_a$ independent of the choice of E. For larger values of k we, however, find a different behavior. In this case $\omega(k,q)$ can be interpreted as a double-well potential and the motion can be stuck within a single-well for sufficiently small energies $E < \omega(k, q = 0)$. In consequence, the relation $q_b = -q_a$ is no longer valid and the motion will no longer be symmetric under the reflection $q \to -q$.
In order to determine the mesonic energy bands $\mathcal{E}(J,k)$ we apply a semiclassical Bohr-Sommerfeld quantization to the set of conjugate variables $(q(t), x(t))$ given by

$$J = \oint \mathrm{d}x\, q(x) = 2\pi\Big(n - \frac{1}{2}\Big) \qquad \text{with} \quad n \in \mathbb{N}. \tag{10}$$

Using the functional form of $q(t)$ and $x(t)$ of (9) we thus find

$$J = 2\chi^{-1}\mathcal{E}(J,k)(q_b - q_a) - 2\chi^{-1}\int_{q_a}^{q_b} \mathrm{d}q\, \omega(k,q) \tag{11}$$

However, due to the Pauli exclusion principle n is forced to be even when the two fermions can come in contact. We thus find two different conditions referring to the cases of a symmetric motion (n even integer) and a motion stuck within a single well of the double-well potential (n integer). For a more detailed derivation of this quantization procedure we refer to Ref. [2]. Solving Supplementary Eq. (11) numerically equips us with all information required to describe the thermodynamics of the prethermal state, as we now discuss.

2. THERMODYNAMICS FOR MESONIC SYSTEMS

In the extremely dilute regime, the typical size of a meson is negligible with respect to their relative distance and their thermodynamics can be approximated as if they were point-like particles. However, mesons are extended objects and their size gives non-neglible contributions moving aside from the extreme dilute scenario. Therefore, we now aim to a better treatment where mesons are considered as extended objects with a fixed length $\ell(J,k)$ which in first approximation can be taken as the average magnetization. Despite the fact this is a rather crude approximation (for example, the size of the meson oscillates in time), it nicely captures features beyond the extreme dilute scenario. Hence, we now discuss the thermodynamics of a gas of hard-rods, where the length of the meson depends on its momentum k and internal energy level J. The momentum k is quantized in units of $2\pi/L$, but we are eventually interested in the thermodynamic limit and replace summations with integrals. Even though we wish to address directly the semiclassical limit, considering the correct momentum quantization is necessary to obtain the correct phase-space normalization of the thermal curves. In the limit of a weakly interacting many-meson system all thermodynamic information is contained in the grand-canonical partition function

$$\mathcal{Z} = \sum_{\{\rho_J(k)\}_{J,k}} e^{\mathcal{S}} e^{-L\beta \sum_{J,k} \rho_J(k)(\mathcal{E}(J,k) - \mu)}\,, \tag{12}$$

where $\rho_J(k)$ is the density of mesons with quantum numbers (J,k) and energies $\mathcal{E}(J,k)$. In this notation, J is the classical action variable that is discretized in units of 2π in the quantum case, due to the Bohr-Sommerfeld quantization condition. The summation over the densities should be interpreted in a path integral sense, but it is useful to think about it as a discrete object first and take the limit at the end. The system size is L. We also introduce the number of mesons with quantum number (J,k) as $N(J,k) = L\mathrm{d}k\rho_J(k)$, with $\mathrm{d}k$ the size of a small momentum cell.

The chemical potential μ ensures the meson number conservation present in the prethermal regime. The summation included in Supplementary Eq. (12) takes into account all possible ways to distribute a given number of mesons in the system. While the

summation over $\{\rho_J(k)\}_{J,k}$ spans the possible populations for each quantum number, the entropic factor $e^{\mathcal{S}}$ counts the possible spatial arrangements of the mesons for a given population distribution. The entropic term is sensitive to the length of the mesons: as anticipated, let $\ell(J,k)$ be the effective length of a meson, which we approximate as a constant. To simplify notation, we group the two quantum numbers in a single one $(J,k) \to \eta$, hence $\ell(J,k) \to \ell_\eta$. This identification takes our system of mesons to be a system of hard-rods of different lengths $\{\ell_\eta\}_\eta$ with N_η particles for each species. For the sake of simplicity, we now choose to neglect the discreteness of the lattice and treat the position of a meson as a continuous variable. This approximation is valid either in the small density limit where finite-volume effects are negligible, or in the limit where mesons are large compared with the unit cell. Therefore, we expect corrections can be important only in the extreme limit of very dense and tight mesons.

To compute the partition function, we first introduce an ordering in the particle species: mesons with quantum number η will be contained in an interval of length L_η, where $L = \sum_\eta L_\eta$. The ordered partition function due to the spatial degrees of freedom is readily computed as (we assume η runs from 1 to m)

$$\mathcal{Z}_m^{(ord)} = \int_0^L \mathrm{d}L_1 \mathrm{d}L_2 \dots \mathrm{d}L_m \, \delta\Big(L - \sum_i L_i\Big) \prod_{r=1}^m \mathcal{Z}_1(L_r, N_r) = \int_{L_1^{(min)}}^{L_1^{(max)}} \mathrm{d}L_1 \int_{L_2^{(min)}}^{L_2^{(max)}} \mathrm{d}L_2 \cdots \int_{L_m^{(min)}}^{L_m^{(max)}} \prod_{r=1}^m \mathcal{Z}_1(L_r, N_r)$$
$$= \frac{1}{(N_1 + N_2 + \dots + N_m)!} \prod_{r=1}^m (L - N_1\ell_1 - N_2\ell_2 - \dots - N_m\ell_m)^{N_r}. \tag{13}$$

Here we introduced the minimal and maximal length of subsystem r as $L_r^{(min)} = N_r \ell_r$ and $L_r^{(max)} = L - \sum_{n<r} L_n - \sum_{n>r} N_n \ell_n$, respectively, to make the constraint of the δ-distribution explicit. The first, hereby, just takes into account that the subsystem r containing N_r particles of length ℓ_r can not be compressed further than $L_r^{(min)}$. The upper bound results from the opposite situation. Having already fixed the subsystem sizes $L_1, ..., L_{r-1}$ we can compress the remaining subsystems to their minimal configuration and find $L_r^{(max)}$. For the last equality of Supplementary Eq. (13) we used the well-known result for the partition function of a simple gas of a single species of hard-rods $\mathcal{Z}_1(L,N) = [(L - N\ell)]^N / N!$. Since the different particles are grouped into subsystems the partition function of Supplementary Eq. (13) does not take into account reordering of the individual meson species. This can, nevertheless, be accounted for by a combinatorial factor

$$\mathcal{Z}_m = \binom{N_1 + N_2 + \dots + N_m}{N_m} \binom{N_1 + N_2 + \dots + N_{m-1}}{N_{m-1}} \dots \binom{N_1 + N_2}{N_2} \mathcal{Z}_m^{(ord)}$$
$$= \prod_{r=1}^m \left(\frac{1}{N_r!}\right) (L - N_1\ell_1 - N_2\ell_2 - \dots - N_m\ell_m)^{N_1 + N_2 + \dots + N_m}. \tag{14}$$

When computing Supplementary Eq. (13), we did not take into account the momentum quantization which allows for a reshuffle of the particles within the single momentum cell of width $\mathrm{d}k$. Due to quantization, such a cell has $\frac{L}{2\pi}\mathrm{d}k$ available quantum numbers. We now consider the problem of arranging N_r indistinguishable particles on $\frac{L}{2\pi}\mathrm{d}k$ sites. We neglect the case of double occupancies, which is equivalent to consider a classical statistics (in contrast with Fermi or Bose statistics). In this case, the number of possible arrangements is $\frac{1}{N_r!}\left(\frac{L\mathrm{d}k}{2\pi}\right)^{N_r} = \frac{L^{N_r}}{N_r!}\left(\frac{\mathrm{d}k}{2\pi}\right)^{N_r}$. Now, we argue that the prefactor L^{N_r} has already been taken into account in Supplementary Eq. (13), while the factor $1/N_r!$ has already been accounted when considering Supplementary Eq. (14). Hence, one is left with the additional phase-space contribution $\left(\frac{\mathrm{d}k}{2\pi}\right)^{N_r}$. We can now finally identify the entropic term as

$$\mathcal{S} = \log\left(\mathcal{Z}_m \prod_r \left(\frac{\mathrm{d}k}{2\pi}\right)^{N_r}\right) \tag{15}$$

In the hypothesis that N_r is large and using a Stirling approximation for the factorial, one finds

$$\mathcal{S}/L \approx \sum_{r=1}^m \mathrm{d}k\rho_r \Big[\ln\Big(\frac{1 - \mathrm{d}k\rho_1\ell_1 - \mathrm{d}k\rho_2\ell_2 - \dots - \mathrm{d}k\rho_m\ell_m}{2\pi\rho_r}\Big) + 1\Big]. \tag{16}$$

By the extensivity of the entropic term, in the thermodynamic limit $L \to \infty$ the partition function localizes to the saddle point of the free energy $\mathcal{F} = -\beta^{-1}\log\mathcal{Z}$

$$-L^{-1}\beta\mathcal{F} = \sum_J \int \mathrm{d}k\rho_J(k)\Big[\ln\Big(1 - \sum_{J'}\int \mathrm{d}q\ell(J',q)\rho_{J'}(q)\Big) - \ln(2\pi\rho_J(k)) + 1 - \beta(\mathcal{E}(J,k) - \mu)\Big] \tag{17}$$

Finally, imposing $\delta\mathcal{F}/\delta\rho_J(k) = 0$ we find

$$\rho_J(k) = \frac{1 - \rho M}{2\pi} \exp\left[-\beta(\mathcal{E}(J,k) - \mu) - \frac{\rho\ell(J,k)}{1 - \rho M}\right] \tag{18}$$

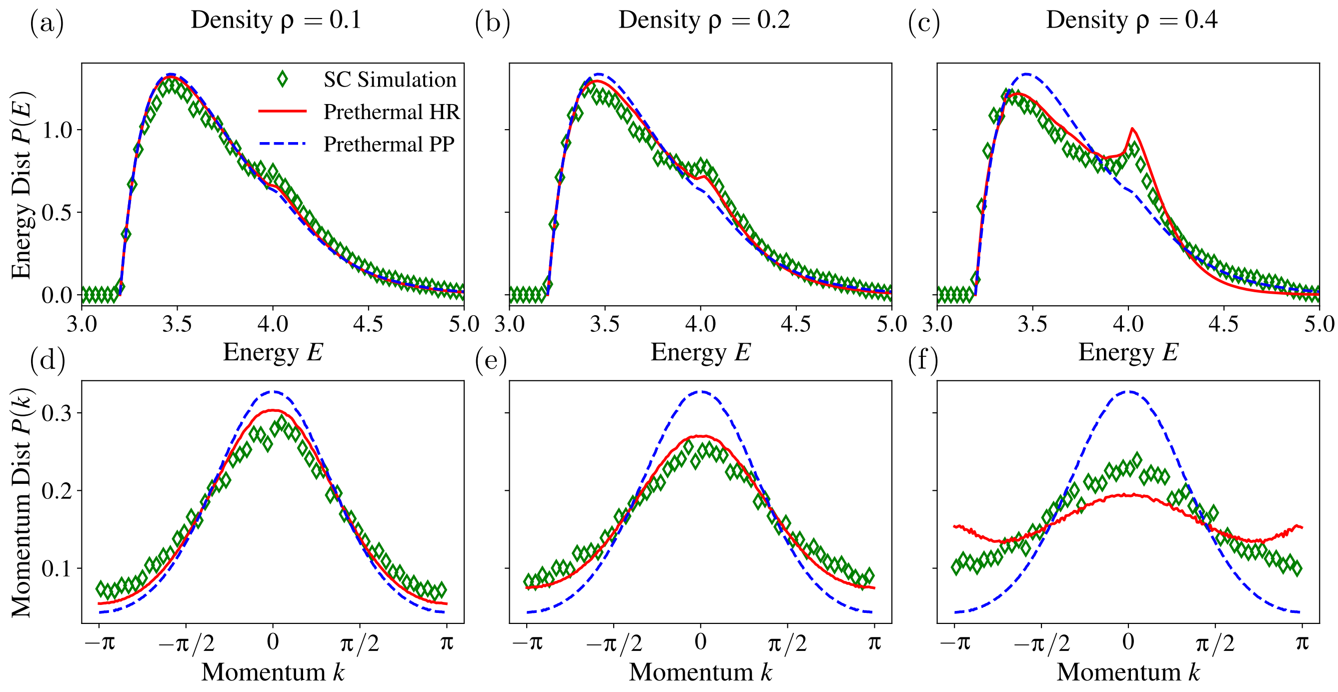

Supplementary Figure 1. **Effect of finite-volume corrections.** We benchmark our predictions from thermodynamic analysis for a system of point particles (PP) as well as for an ensemble of hard-rods (HR) against the results of semiclassical simulations. (a)-(c) We display the energy distribution $P(E)$ of the prethermal steady state for different densities of mesons ρ in a system characterized by a longitudinal and transverse field $(h_{\parallel}, h_{\perp}) = (0.015, 0.2)$. The initial energy per meson is thereby on average given by $E = 3.73737$. We find that the finite-volume correction included in the HR treatment (red solid curves) improves the thermodynamic description of the semiclassical data points (green markers) compared to the PP description (blue dashed curves). (d)-(f) The prethermal state of hard rods captures the momentum distribution $P(k)$ especially well for densities $\rho \lesssim 0.2$. For very high densities $\rho \approx 0.4$ deviations become apparent.

Where the meson coverage ρM, the total density ρ and the mean energy E are

$$\text{(i)} \qquad \rho = \sum_J \int \mathrm{d}k\, \rho_J(k) \tag{19}$$

$$\text{(ii)} \qquad E = \sum_J \int \mathrm{d}k\, \mathcal{E}(J,k)\rho_J(k) \tag{20}$$

$$\text{(iii)} \qquad \rho M = \sum_J \int \mathrm{d}k\, \ell(J.k)\rho_J(k). \tag{21}$$

The meson density and coverage must be computed self consistently with Supplementary Eq. (18). On prethermal states, thus enforcing the meson density conservation, the chemical potential μ and inverse temperature β must be chosen to match the initial density ρ and energy E. On the other hand, thermal ensembles fix only the energy E and $\mu = 0$. It is worth to consider the momentum-energy probability distribution of a meson $P(E,k)$ in the classical case. We start considering $P(E,k)$ in the quantum regime writing it as

$$P(E,k) = \sum_J \rho_J(k)\delta(E - \mathcal{E}(J,k)), \tag{22}$$

with δ a Dirac delta. In the semiclassical regime, we can replace the summation over J with an integral $\sum_J \to \int \frac{\mathrm{d}J}{2\pi}$, where the factor 2π comes from the Born-Sommerfeld quantization condition $J = 2\pi(n - 1/2)$

$$P(E,k) = \frac{1-\rho M}{(2\pi)^2} e^{-\beta(E-\mu)} \int \mathrm{d}J \delta(E - \mathcal{E}(J,k)) e^{-\frac{\rho \ell(J,k)}{1-\rho M}}. \tag{23}$$

Notice that in the small density limit $\rho \to 0$, the coverage ρM vanishes as well and one recovers the usual thermal distribution of non-interacting particles.

Finally, we wish to explicitly discuss how $\ell(J,k)$ is computed: as we already mention, we estimate the length of the hard rod approximation of the meson with its average length. In the classical limit, this amounts to computing the time average of the

distance between the two fermions within one period of the oscillation. In the quantum regime, instead, $\ell(J,k)$ is computed by considering the quantum expectation value of the relative distance $|x|$ (see Supplementary Eq. (8)) on the energy eigenstate. Of course, the two definitions coincide in the semiclassical limit.

To further emphasize the importance of the finite volume corrections, we refer to Supplementary Fig. 1 providing a benchmark of our thermodynamic predictions against semiclassical simulations. The semiclassical data for the energy distribution $P(E)$ of the prethermal steady state shown in Supplementary Fig. 1 (a) - (c) reveals that systems of higher meson densities ρ in fact exhibit increased meson occupancy towards the edges of the Brillouin zone ($E \approx 4.0$), where mesons show their smallest average lengths. While the conventional thermodynamic ansatz for point particles fails in reproducing this feature of the steady state, including finite volume corrections indeed allows us to capture this effect. This is further supported by results for the momentum distributions $P(k)$ of the same systems, as illustrated in Supplementary Fig. 1 (d) - (f).

3. INITIALIZING MOVING MESONS BY MODULATED PULSES OF THE TRANSVERSE FIELD

The prethermalization time scale can be strongly reduced when mesons are initialized with a finite velocity, as we demonstrated in Fig. 2. This scenario can be achieved through a different state preparation, by replacing the homogeneous quench in the transverse field with a modulated pulse on a period of n sites [5], i.e., $\hat{H}_{\text{Pulse}} = -\delta(t)\sum_j \mathrm{h}_j\sigma_j^x$, where $\mathrm{h}_{j+n} = \mathrm{h}_j$. While fermions are still excited in pairs (k,k'), the periodic modulation breaks translation invariance thus giving a non-trivial momentum to the meson $k+k' = 2\pi j/n$, with j an integer. While this strategy allows us to maintain the dilute meson approximation, it creates moving mesons right from the beginning, thus promoting scrambling. In particular, let us assume the state is initially prepared in the ground state $|0\rangle$ of the transverse-field Ising model $\hat{H}_{\text{tr}} = -\sum_j \sigma_{j+1}^z\sigma_j^z + h_\perp\sigma_j^x$, then we consider a pulse Hamiltonian in the form $\hat{H}_{\text{Pulse}} = -\delta(t)\sum_j \mathrm{h}_j\sigma_j^x$, with h_j being a periodic modulation with period n, i.e. $\mathrm{h}_j = \mathrm{h}_{j+n}$. After the pulse application, the state evolved into $|\psi\rangle \equiv e^{i\sum_j \mathrm{h}_j\sigma_j^x}|0\rangle$ which we now characterize. After the pulse, the longitudnal field is activated and $|\psi\rangle$ is used as the initial state for the confining dynamics: we assume the regime of weak confinement, hence we neglect meson production caused by the activation of the longitudinal field $h_\parallel$. As a first step, we express the pulse in the fermionic basis and eventually in the modes of the transverse Ising Hamiltonian, by Supplementary Eq. (2). For the sake of a more compact notation, we define the non-rotated fermionic modes in the Fourier basis as $\hat{\alpha}(k) = \cos\theta_k\hat{\gamma}(k) + i\sin\theta_k\hat{\gamma}^\dagger(-k)$, where $\{\hat{\alpha}(k),\hat{\alpha}^\dagger(q)\} = \delta(k-q)$. We write the pulse as

$$-\sum_j \mathrm{h}_j\sigma_j^x = \sum_j 2\mathrm{h}_j c_j^\dagger c_j + \text{const.} = \int_{-\pi}^{\pi}\frac{\mathrm{d}k\mathrm{d}q}{2\pi}\left[\sum_j 2\mathrm{h}_j e^{ij(k-q)}\right]\hat{\alpha}^\dagger(q)\hat{\alpha}(k) + \text{const.} =$$
$$\int_{-\pi}^{\pi}\mathrm{d}k\mathrm{d}q\,2\tilde{\mathrm{h}}(k-q)\delta(e^{in(k-q)}-1)\hat{\alpha}^\dagger(q)\hat{\alpha}(k) + \text{const.} = \sum_{j,j'}\int_{-\pi/n}^{\pi/n}\mathrm{d}k\,\frac{2}{n}\tilde{\mathrm{h}}(2\pi(j-j'))\hat{\alpha}^\dagger(k+2\pi j'/n)\hat{\alpha}(k+2\pi j/n) + \text{const.}\,. \tag{24}$$

Above, we then use the periodicity of h_j to extract a delta function in the momentum space and defined $\tilde{\mathrm{h}}(k) \equiv \sum_{j=0}^{n-1} e^{ikj}\mathrm{h}_j$. We now use this expression to show that

$$|\psi\rangle = e^{i\sum_j \mathrm{h}_j\sigma_j^x}|0\rangle = \mathcal{N}\exp\left[\frac{1}{2}\sum_{jj'}\int_{-\pi/n}^{\pi/n}\mathrm{d}k\,\mathcal{M}_{j,j'}(k)\hat{\gamma}^\dagger(k+j2\pi/n)\hat{\gamma}^\dagger(-k-j'2\pi/n)\right]|0\rangle\,. \tag{25}$$

Above, $\mathcal{M}_{j,j'}(k)$ is a $k-$dependent $n\times n$ complex matrix. Notice that, due to the fermionic anticommutation relations, it holds $\mathcal{M}_{j,j'}(k) = \mathcal{M}_{n-j',n-j}(-k)$. Supplementary Eq. (25) generalizes the state of Supplementary Eq. (4) to the case of fermions pairwise excited, but with pairs possibly having non-zero total momentum.

To show Supplementary Eq. (25) it is convenient to introduce a fictitious parameter λ and considering $|\psi_\lambda\rangle = e^{i\lambda\sum_j \mathrm{h}_j\sigma_j^x}|0\rangle$, by showing infinitesimal $\lambda-$changes move within the parametrization (25) with a $\lambda-$ dependent wavefunction $\mathcal{M}_{jj'}^\lambda(k)$ and normalization $\mathcal{N}_\lambda$. By introducing the $\lambda-$dependence in Supplementary Eq. (25) and taking the derivative, one obtains

$$\partial_\lambda|\psi_\lambda\rangle = \left(\frac{\partial_\lambda\mathcal{N}_\lambda}{\mathcal{N}_\lambda} + \frac{1}{2}\sum_{jj'}\int_{-\pi/n}^{\pi/n}\mathrm{d}k\partial_\lambda\mathcal{M}_{j,j'}^\lambda(k)\hat{\gamma}^\dagger(k+j2\pi/n)\hat{\gamma}^\dagger(-k-j'2\pi/n)\right)|\psi_\lambda\rangle\,. \tag{26}$$

On the other hand, by the very definition one has $\partial_\lambda|\psi_\lambda\rangle = i\sum_j \mathrm{h}_j\sigma_j^x|\psi_\lambda\rangle$. By repeatedly using the fermionic commutation relation on Supplementary Eq. (25), the action of $\hat{\alpha}(k+2\pi j/n)$ on $|\psi_\lambda\rangle$ is computed as

$$\hat{\alpha}(k+2\pi j/n)|\psi_\lambda\rangle = \left[\sum_s \cos\theta_{k+2\pi j/n}\mathcal{M}_{j,s}^\lambda(k)\hat{\gamma}^\dagger(-k-s2\pi/n) + i\sin\theta_{k+2\pi j/n}\hat{\gamma}^\dagger(-k-2\pi j/n)\right]|\psi_\lambda\rangle\,. \tag{27}$$

Further applying $\hat{\alpha}^\dagger(k+2\pi j'/n)$ on the so-obtained state, one can proceed by the same methods and the action of Supplementary Eq. (24) on $|\psi_\lambda\rangle$ is easily obtained. After some simple, but tedious calculations it is shown that the gaussian form of Supplementary Eq. (25) closes the equations if $\mathcal{M}^\lambda_{j,j'}$ satisfies

$$i\partial_\lambda \mathcal{M}^\lambda_{j,j'}(k) = \sum_{s,s'} \frac{2}{n}\tilde{\mathrm{h}}(2\pi(s-s'))\left[\cos\theta_{k+2\pi s/n}\mathcal{M}^\lambda_{s,j}(k) + i\sin\theta_{k+2\pi s/n}\delta_{j,s}\right]\left[-i\sin\theta_{k+2\pi s'/n}\mathcal{M}^\lambda_{s',j'}(k) + \cos\theta_{k+2\pi s'/n}\delta_{j',s'}\right] + \left[k\to -k\,, j\to n-j'\,, j'\to n-j\right], \quad (28)$$

where the term $[k\to -k\,, j\to n-j'\,, j'\to n-j]$ is obtained from the first through the proper index replacement. Finally, the desired matrix $\mathcal{M}_{j,j'}(k)$ is computed by integrating the above equation up to $\lambda=1$. Albeit a close analytical expression is hard to obtain, the $n\times n$ matrix relation of Supplementary Eq. (24) can be easily numerically integrated. Furthermore, in the problem at hand we are mostly interested in the regime of dilute mesons, i.e. when h_j is small. Supplementary Eq. (28) is easily solved at the leading order in h_j obtaining

$$i\mathcal{M}^\lambda_{j,j'}(k) = \frac{2}{n}\tilde{\mathrm{h}}(2\pi(j-j'))i\sin\theta_{k+2\pi j/n}\cos\theta_{k+2\pi j'/n} + \frac{2}{n}\tilde{\mathrm{h}}(2\pi(j'-j))i\sin\theta_{-k-2\pi j'/n}\cos\theta_{-k-2\pi j/n} + \mathcal{O}(\mathrm{h}^2)\,. \quad (29)$$

4. LATTICE GAUGE THEORIES: THE EXAMPLE OF THE U(1) QUANTUM LINK MODEL

As an example how the proposed multistage thermalization mechanism generalizes to lattice gauge theories we want to discuss the physics of a $\mathrm{U}(1)$ quantum link model (QLM)

$$H_{\mathrm{QLM}} = -\omega\sum_{j=1}^{L-1}(\phi_j^\dagger S^+_{j,j+1}\phi_{j+1} + \mathrm{h.c.}) + \frac{m}{2}\sum_{j=1}^{L}(-1)^j\phi_j^\dagger\phi_j - 2h_\parallel\sum_{j=1}^{L-1}S^z_{j,j+1}, \quad (30)$$

where staggered Kogut-Susskind fermionic matter [6, 7] is described by the creators $\phi_j^\dagger$ and annihilators ϕ_j and we additionally include gauge degrees of freedom encoded by a local set of spin-$\frac{1}{2}$ operators $\{S^z_{j,j+1}, S^+_{j,j+1}, S^-_{j,j+1}\}$ acting on the links $(j,j+1)$. The content of fermionic excitations in the model in this staggered convention can be counted using a generalized number operator $n_j = \frac{1}{2}[1-(-1)^{\phi_j^\dagger\phi_j + j}]$. This relates to the picture that holes located at odd sites can be interpreted as a antiquark $(\bar{q})$, whereas a particle located at a even site resembles a quark (q): in the regime where m is infinite, the ground state of the theory is readily identified with the vacuum of the theory $|\mathrm{Vac}\rangle$ (where quarks and antiquark are absent). For large, but finite mass m, bare particles must be suitably renormalized, but the picture remains qualitatively the same: for the sake of clarity, we will mainly refer to the large m limit.

In $(1+1)$-dimensional lattice gauge theory Gauss law imposed by local gauge symmetry on each site completely determines the associated configuration of the gauge field after choosing its values at the boundaries and the desired gauge sector. The latter is defined via the generators of the gauge symmetry

$$G_j = S^z_{j,j+1} - S^z_{j-1,j} - \phi_j^\dagger\phi_j + \frac{1-(-1)^j}{2}. \quad (31)$$

The set of $\{G_j\}_j$ commutes with the Hamiltonian, i.e. $[H, G_j] = 0\ \forall j$, and impose a splitting of the Hilbert space in gauge sectors characterized by $G_j|\psi\rangle = g_j|\psi\rangle$ and labeled by the eigenvalues g_j. Within the zero charge sector $g_j = 0$, the vacuum state for the matter is associated with a ferromagnet in the gauge spin degrees of freedom, where $S^z_{j,j+1}$ is aligned in the same direction. For $h_\parallel > 0$, the ground state has positive magnetization $\langle S^z_{j,j+1}\rangle = +1/2$. If one now introduces matter particles in the state, the Gauss law forces the gauge spin to change sign each time a quark (or antiquark) is crossed. Hence, quark-antiquark pairs will be separated by gauge spins pointing in the wrong direction: a linear potential proportional to $h_\parallel$ is thus established between quark-antiquark pairs, akin to what happens between fermions in the Ising spin chain. We have seen that in our (1+1)-dimensional QLM fermionic excitations and the configuration of the gauge field are closely related with each other via Gauss law. Therefore, we can simply integrate out the fermionic content of our theory within a given gauge sector and end up in a pure $(1+1)$-dimensional gauge theory. To better highlight the physics of confinement, we wish to address the effective Hamiltonian in the large mass limit, with the help of Supplementary Fig. 2.

For this we define the rescaled QLM Hamiltonian as

$$\tilde{H}_{QLM} = \sum_{j=1}^{L}(-1)^j\phi_j^\dagger\phi_j - \frac{4h_\parallel}{m}\sum_{j=1}^{L-1}S^z_{j,j+1} - \frac{2\omega}{m}\sum_{j=1}^{L-1}(\phi_j^\dagger S^+_{j,j+1}\phi_{j+1} + \mathrm{h.c.}) \equiv \tilde{H}^0 + \tilde{H}^1. \quad (32)$$

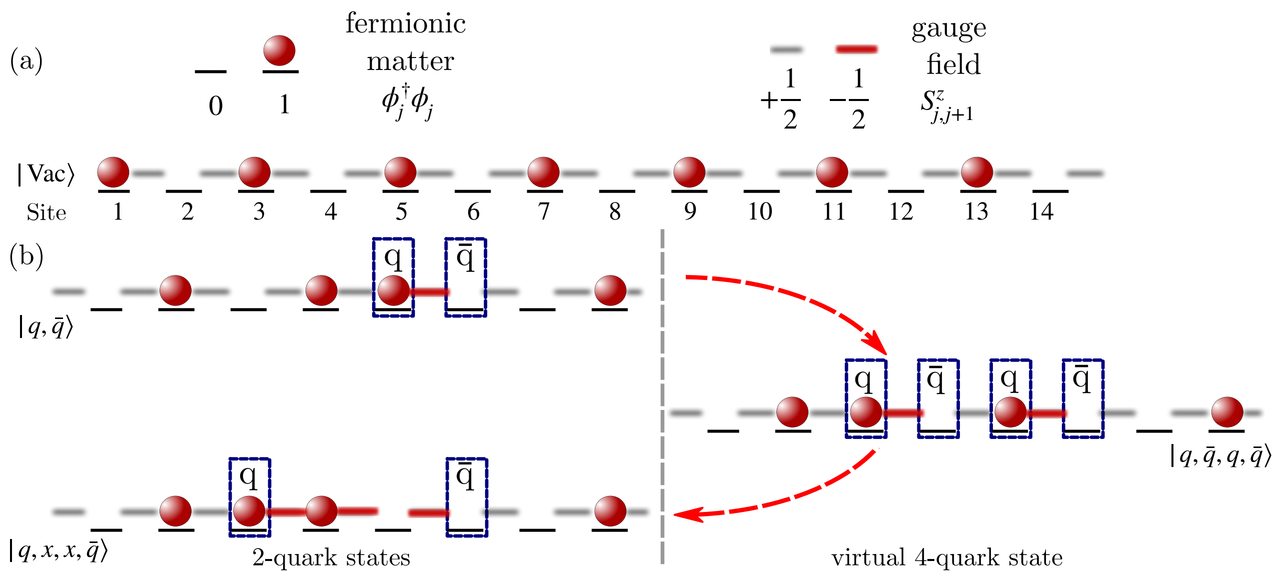

Supplementary Figure 2. **Low-energy spectrum of QLM in gauge sector** $g_j = 0$**.** (a) We show the vacuum configuration $|\mathrm{Vac}\rangle$ of the $\mathrm{U}(1)$ quantum link model with staggered fermions. Fermionic matter associated to creators $\phi_j^\dagger$ is located on odd sites and the gauge field takes values of $\langle S^z_{j,j+1}\rangle = \frac{1}{2}$ on the links. (b) Application of the gauge-matter coupling of H_{QLM} allows for creation of quark-antiquark pairs as emphasized in configuration $|q,\bar{q}\rangle$. Dynamical processes of the fermionic excitations (q and $\bar{q}$) can appear via virtual pair-creation processes linking a four-quark configuration $|q,\bar{q},q,\bar{q}\rangle$ to a two-quark state with larger distance of fermionic excitations $|q,x,x,\bar{q}\rangle$.

Whereas Hamiltonian Supplementary Eq. (32) itself does not contain a dynamical term for a single quark, dynamics naturally appears in second order perturbation theory via an intermediate virtual pair-creation process $\mathcal{O}(\frac{\omega}{m})$. In this picture we can go from a configuration $|q,\bar{q}\rangle$ of the particle-antiparticle pair to a configuration with larger distance between the composite particles $|q,x,x,\bar{q}\rangle$ via virtual transition to a four-quark configuration $|q,\bar{q},q,\bar{q}\rangle$, as emphasized in Supplementary Fig. 2 (b). Notice that we only find a two-site hopping process finally resulting from the staggered nature of the vacuum state. To write the final effective Hamiltonian in a more compact form, it is useful to introduce the following mapping [7] between the gauge degrees of freedom and new spin variables

$$\sigma_j^z \Longleftrightarrow (-1)^j 2S^z_{j,j+1}\,, \qquad \sigma_j^x \Longleftrightarrow (\phi^\dagger_{j-1}S^+_{j-1,j}\phi_j + \mathrm{h.c.})\,. \tag{33}$$

In this notation, the effective Hamiltonian reads

$$H^{\mathrm{eff}} = \sum_{j=1}^{L-2}\sigma_j^z\sigma_{j+1}^z - h_\perp^{\mathrm{eff}}\sum_{j=1}^{L-2}\sigma_j^x\sigma_{j+1}^x - \frac{2h_\parallel}{m}\sum_{j=1}^{L-1}(-1)^j\sigma_j^z. \tag{34}$$

Where the effective transverse field of Supplementary Eq. (34) is determined as

$$-h_\perp^{\mathrm{eff}} = \sum_{|\nu\rangle}\frac{\langle q_{j-2},x_{j-1},x_j,\bar{q}_{j+1}|\tilde{H}^1|\nu\rangle\langle\nu|\tilde{H}^1|q_j,\bar{q}_{j+1}\rangle}{E^0_{|q_j,\bar{q}_{j+1}\rangle}-E^0_{|\nu\rangle}} = -\frac{4\omega^2}{m}\Big[1+\frac{4h_\parallel}{m^3}\Big]. \tag{35}$$

Above, we used that the unperturbed energy difference between both states is, moreover, given by $E^0_{|q,\bar{q}\rangle} - E^0_{|q_{j-2},\bar{q}_{j-1},q_j,\bar{q}_{j+1}\rangle} = -\big[m + \frac{4h_\parallel}{m}\big]$.

This precisely resembles an antiferromagnetic version of our spin toy model. The energy cost of a ferromagnetic domain wall thereby directly relates to the mass of a quark excitation in the rescaled QLM of Supplementary Eq. (32) and the confining coupling of the gauge field translates to interaction with an external staggered longitudinal field $(-1)^j 2h_\parallel/m$. Having obtained the effective spin Hamiltonian of Supplementary Eq. (34) we can proceed with quantizing the energy levels of meson-like $(q,\bar{q})$ excitations in similar manner as we did for the Ising chain in previous sections. In the limit of large m, where the Ising energy scale dominates contributions of the remaining terms we can once more restrict our considerations to the two particle sector and find a Hamiltonian

$$H^{\mathrm{AFM}}_{\mathrm{2pt}} = \int \mathrm{d}k_1\mathrm{d}k_2[\tilde{\epsilon}(k_1)+\tilde{\epsilon}(k_2)]|k_1,k_2\rangle\langle k_1,k_2| + \sum_{j_1,j_2}\frac{8h_\parallel}{m}|j_1-j_2||j_1,j_2\rangle\langle j_1,j_2| \tag{36}$$

similar to Supplementary Eq. (6) for the two-particle problem in the initial Ising model, where we choose to write down the confining part of Supplementary Eq. (36) in its real space representation. The kinetic part diagonal in the basis of momentum states $|k_1, k_2\rangle$, however, now contains the dispersion law of the antiferromagnetic Ising chain ($\tilde{\epsilon}(k) \approx 2 - 2h_\perp^{\rm eff}\cos(2k) + \dots$). With this we can proceed in analogous fashion to section (1) and obtain the energy bands for the mesonic exciations using suited variations of Supplementary Eqs. (8) to (11).

Implementation in Rydberg atoms arrays.— As pointed out in Ref. [7], the Quantum Link Model finds a natural implementation in Rydberg atoms arrays. To connect the two setups, one relies once again on the mapping of Supplementary Eq. (33), but this time we will not use perturbation theory and directly map the Hamiltonian of Supplementary Eq. (30) to the Fendley-Sengupta-Sachdev Hamiltonian [8] with an additional staggered field

$$H_{\rm Ryd} = \mathcal{P}\left[\sum_j -\omega\sigma_j^x - \frac{m}{2}\sigma_j^z + h_\parallel(-1)^j\sigma_j^z\right]\mathcal{P}\,. \tag{37}$$

Where the ground state and Rydberg excited state of each atom are respectively denoted with the spin down and up in the $z-$basis. Above, the projector $\mathcal{P}$ enforces the Rydberg blockade, namely two nearby atoms cannot be simultaneously excited due to energetic constraints, which is a direct consequence of the Gauss law of the original Quantum Link Model. As we discussed, in the large mass limit the vacuum of the gauge theory in the zero charge sector consists in a $z-$ferromagnet in the gauge field. Therefore, through the mapping of Supplementary Eq. (33), this state is mapped to a Rydberg configuration where atoms are excited on alternating bonds, the weak staggered coupling $\propto h_\parallel$ breaks the $\mathbb{Z}_2$ symmetry favoring one staggered configuration in place of the companion related by a one-site shift. Quark-antiquark pairs are in correspondence with defects in this staggered configuration: by acting on the vacuum with a weak rotation along the $x-$direction $R = e^{i\theta\sum_j\sigma_j^x}$ with $\theta \ll 1$ it is possible to create a homogeneous gas of quark-antiquark pairs with zero total momentum, akin of the initial state produced in the Ising chain. Such an excited state will then relax through the multistage thermalization dynamics we exhaustively discussed in the Ising chain.

5. EXACT DIAGONALIZATION IN THE FEW KINKS SUBSPACE

Building on the stability of the fermions for exponentially long times, one can project the Hamiltonian of Supplementary Eq. (6) within the few-fermions sector. In this way, by exact numerical integration of the few-fermion wavefunction, we can access very long timescales and explore prethermalization.

While this strategy can be applied for arbitrary values of the transverse field by considering Supplementary Eq. (6) (generalized to many fermions), here, we focus on the small transverse field limit where the Hamiltonian is further simplified. In this regime, the kinetic part reduces to nearest-neighbor hopping and the fermions are equivalently describing domain walls. Hence, let $\Psi(j_1, j_2, ..., j_{2n-1}, j_{2n})$ be the wave function labeling the state with domain walls between the lattice sites $j_i - 1$ and j_i and, without loss of generality, we consider the ordering $j_1 < j_2 < ... < j_{2n}$, and periodic boundary conditions are assumed. Furthermore, we consider the false vacuum to be between the kinks j_{2i-1} and j_{2i}. On this wave funtion, the Hamiltonian acts as

$$[\hat{H}_{\rm Kinks}\Psi](j_1, ..., j_{2n}) = \sum_{i=1}^{2n} -h_\perp[\Psi(j_1, ..., j_i + 1, ..., j_{2n}) + \Psi(j_1, ..., j_i - 1, ..., j_{2n})] + \sum_{i=1}^{n}\chi|j_{2i-1} - j_{2i}|\Psi(j_1, ..., j_{2n})\,. \tag{38}$$

Above, we neglect an overall unimportant constant and the hopping term should respect the hard core constraint $j_1 < j_2 < ... < j_{2n}$. Since we are mostly interested in the scattering among mesons, we consider a translational invariant scenario: this allows us to further enhance the performance of the approach by removing a degree of freedom. For the sake of simplicity, we consider the case of global zero momentum, but the same method can be applied to the general case. It is convenient to use the position of the first domain wall as a reference coordinate and introduce new variables $s_i = j_{i+1} - j_1$. We denote with $\Phi(s_1, ..., s_{2n-1})$ the wavefunction in the relative coordinates. In this case, the dynamics is

$$\begin{aligned}[\hat{H}_{\rm Kinks, zero\ momentum}\Phi](s_1, ..., s_{2n-1}) = -h_\perp[\Phi(s_1 + 1, ..., s_{2n-1} + 1) - \Phi(s_1 - 1, ..., s_{2n-1} - 1)] + \\ \sum_{i=1}^{2n-1} -h_\perp[\Phi(s_1, ..., s_i + 1, ..., s_{2n-1}) + \Phi(s_1, ..., s_i - 1, ..., s_{2n-1})] + \sum_{i=0}^{n-1}\chi|s_{2i+1} - s_{2i}|\Phi(s_1, ..., s_{2n-1})\,.\end{aligned} \tag{39}$$

Above, the first term accounts for the hopping of the first domain wall, which equivalently shift of one site all the relative distances s_i. From the knowledge of the wavefunction Φ, several observables of interest can be computed. First, the total

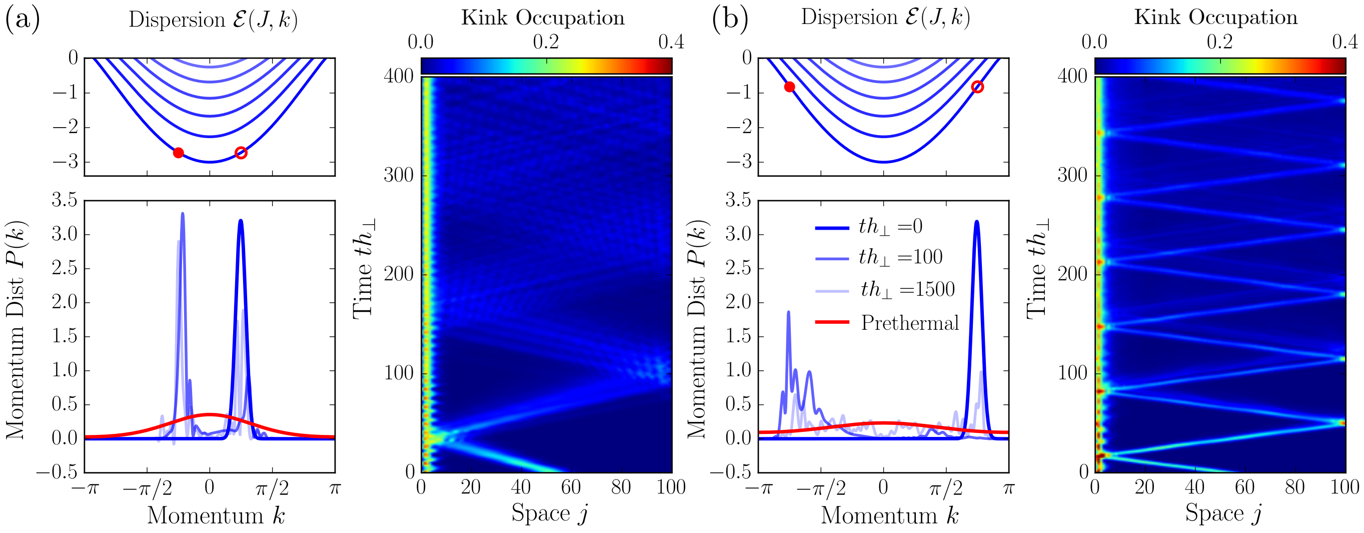

Supplementary Figure 3. **Two-meson dynamics.** Analogously to the three-meson scenario considered in the main text, we illustrate the results for dynamics of two mesons in a system of $L = 100$ sites and confinement field $h_\parallel/h_\perp = 0.1$. We consider two distinct initial states, where the mesons are initialized with energies below (a) or above (b) the second band of the single meson spectrum $\mathcal{E}(J,k)$. The momentum distribution $P(k)$ of the meson with positive initial momentum is evaluated (empty dot in the plot of dispersion bands). (a) A double-peak structure centered at the initial momenta of the mesons $k_0 = \pm\pi/4$ survives even at late times. This characteristics disappears in (b) for initial meson momenta of ($k_0 = \pm 3\pi/4$), where after a time transient various momenta are be occupied. Crucially, for both initial states, (a) and (b), the system does not attain the prethermal state (red line) because scattering events involve only two mesons. This fact clearly distinguishes the scenario of two mesons from the case of three mesons discussed in the main text.

magnetization is directly connected to the length of the mesons, since the part of the chain enclosed within one meson lays in the false vacuum

$$\sum_{j=1}^{L}\langle S_j^z\rangle = L/2 - \sum_{\{s_i\}}\left[s_1 + \sum_{i=1}^{n-1}|s_{2i+1}-s_{2i}|\right]|\Phi(s_1,\dots,s_{2n-1})|^2\,. \tag{40}$$

However, much more information is contained in the probability distribution of the meson length

$$\mathcal{P}_{\text{Length}}(\ell) = \frac{1}{N_{\text{mes}}}\sum_{\{s_i\}}\left[\delta(\ell - s_1) + \sum_{i=1}^{n-1}\delta(\ell - |s_{2i+1}-s_{2i}|)\right]|\Phi(s_1,\dots,s_{2n-1})|^2\,, \tag{41}$$

where δ is a Kronecker delta distribution. Nonetheless, our primary tool to assess prethermalization is the momentum distribution of the mesons. In this case, particular care should be taken when passing from the original coordinates to the relative ones. Let us consider the density matrix in the momentum space defined as

$$\rho(k_1,k_2,\dots|q_1,q_2,\dots) = \sum_{\{j_i\},\{j'_i\}} e^{i\sum_i(k_i j_i - q_i j'_i)}\Psi(j_1,j_2,\dots)\Psi^*(j'_1,j'_2,\dots)\,. \tag{42}$$

Then, we wish to target the momentum distribution of the first meson $P(k)$, defined as

$$P(k) = \sum_{\{k_i\}}\delta(k_1+k_2-k)\rho(k_1,k_2\dots|k_1,k_2,\dots) = \int\frac{\mathrm{d}\omega}{2\pi}\sum_{\{k_i\}}e^{i\omega(k_1+k_2-k)}\rho(k_1,k_2\dots|k_1,k_2,\dots)\,. \tag{43}$$

The integral representation of the Dirac δ distribution is particularly convenient for carrying out the straightforward but lengthy calculations. By plugging the definition of Supplementary Eq. (42) in the above equation and, within the total zero momentum sector, passing to the relative coordinates one finally obtains

$$P(k) = \sum_{\{s_i\}_{i=1}^{2n}} e^{iks_1}\Phi(s_2-s_1,s_3-s_1,\dots,s_{2n}-s_1)\Phi^*(s_2-s_1,s_3,\dots,s_{2n})\,. \tag{44}$$

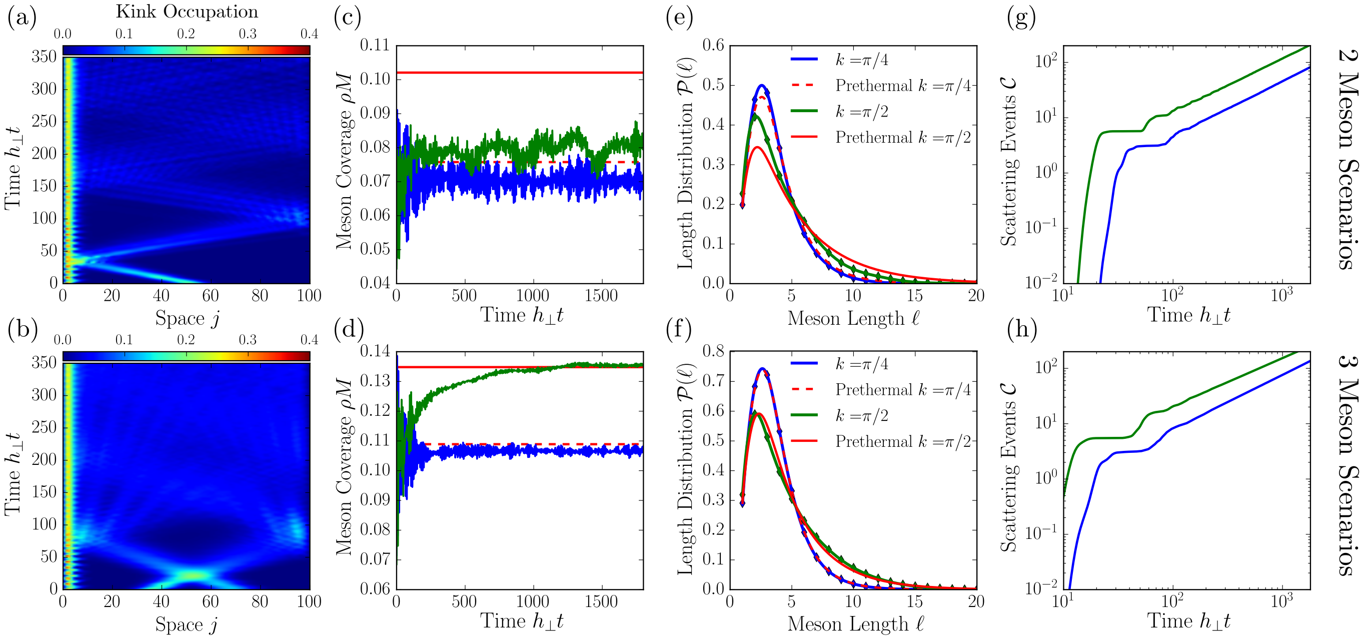

Supplementary Figure 4. **Comparison of two and three meson scenario.** Dynamics in a system of $L = 100$ sites containing (a) two mesons with momenta $k_0 = \pm\pi/2$ and (b) three mesons with momenta $k_0 = 0, \pm\pi/2$, respectively. The underlying confinement field is $h_\parallel/h_\perp = 0.1$. The meson coverage ρM reveals a fundamental difference between systems of (c) two and (d) three mesons. Whereas an ensemble of three mesons relaxes to a prethermal configuration (red dashed and solid lines) provided the initial energy per meson is above the second band of the single meson spectrum, we solely find relaxation to a non-thermal state for the two meson system. (e) - (f) This observation is supported by studies of the length distribution of mesons $\mathcal{P}(\ell)$. (g) - (h) Even though the meson densities are different in the two cases, the number of scattering events is similar. Therefore, an absence of thermalization in the two-meson scenario, due to a reduced number scattering events can be ruled out.

In the main text, we use the momentum distribution to analyze relaxation and prethermalization of initial wavepacket configurations. To this end, we initialize states with two ($n = 2$) or three mesons ($n = 3$) in the form of gaussian wavepackets with tunable initial momenta. The functional form of the initial meson state reads

$$\Phi(s_1, ..., s_{2n}) = \prod_{i=0}^{n-1} \left\{ \phi_{K_i}(s_{2i+1} - s_{2i}) e^{iK_j(\frac{s_{2i+1}+s_{2i}}{2})} W_{\sigma,\bar{X}_i}(s_{2i}, s_{2i+1}; s_{2i+2}, s_{2i+3}) \right\}, \tag{45}$$

where above $W_{\sigma,X}$ is a gaussian wavepacket for the relative distance between to consecutive mesons

$$W_{\sigma,X}(s_{2i}, s_{2i+1}; s_{2i+2}, s_{2i+3}) \propto \exp\left[-\frac{\left((s_{2i} + s_{2i+1}) - (s_{2i+2} + s_{2i+3}) - \bar{X}\right)^2}{\sigma^2} \right] \tag{46}$$

and the wavefunction $\phi_K(s)$ is the mesonic wavefunction of the lowest dispersion band obtained by numerically diagonalizing Supplementary Eq. (38) in the two-fermion sector with total momentum K. Furthermore, we insert a cutoff $\phi_K(|s| < \lambda_c) = 0$ (and similarly in the gaussian wavepackets) to ensure the initial state is correctly ordered. We checked our results to be cutoff-independent.

Further results of the scattering dynamics are provided in supplementary figures 3 and 4.

1. Benchmarking exact diagonalization in the few kinks subspace with tensor networks

After discussing the basics of both exact diagonalization in the few kinks subspace and tensor network simulations it proofs useful to compare both methods with each other to benchmark the accuracy of exact diagonalization results. Since we refer to results of few kinks ansatz in the main text for situations considering up to 3 mesons in the system we would like to test evolution containing $N \in \{1, 2, 3\}$ mesons. As one of the main assumptions leading to the formulation of the few kinks ansatz, moreover, was the sharp nature of fermionic domain wall excitations, the accuracy will crucially depend on the chosen transverse field $h_\perp$ dressing the sharp fermionic kinks. For this reason we also provide simulation results for different choices of $h_\perp \in$

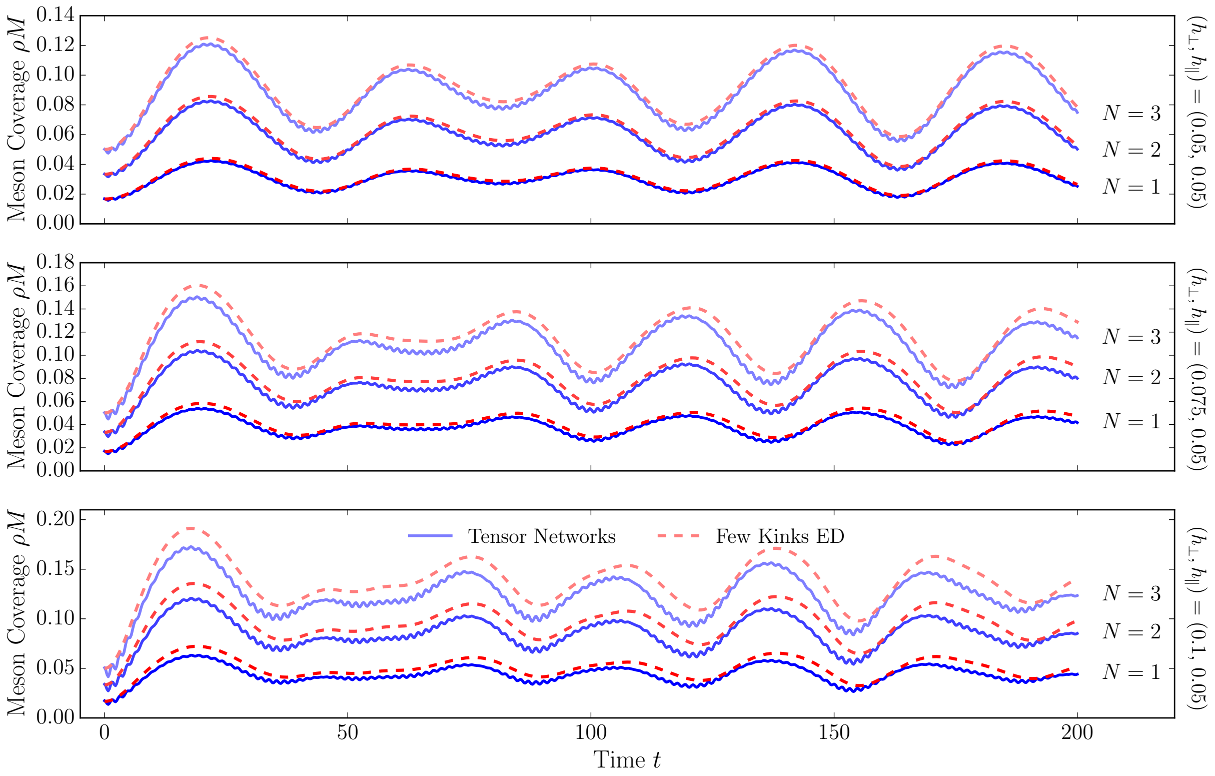

Supplementary Figure 5. **Comparison of few-kink subspace evolution with tensor network predictions.** We benchmark the evolution results for a N meson initial state $|\psi_i^{(N)}\rangle$ obtained using exact diagonalization in the few kink subspace against numerically exact results from tensor network evolution. We consider values for the transverse field of $h_\perp \in \{0.05, 0.075, 0.1\}$ and fixed longitudinal field $h_\parallel = 0.05$ while numbers of $N \in \{1, 2, 3\}$ mesons in the chain are tested. We find very good agreement between results in systems of $L = 60$ sites and bond dimensions up to $\chi = 256$ for small values of $h_\perp = 0.05$. Considering larger values of the transverse field $h_\perp = 0.1$ we find qualitative features of the tensor network evolution to be reproduced by exact diagonalization results, while still differing by a offset expected from dressing of the fermionic domain walls at larger values of $h_\perp$.

$\{0.05, 0.075, 0.1\}$. The value of the longitudinal field component $h_\parallel$ will thereby be kept constant with at a rather small value of $h_\parallel = 0.05$ to guarantee conservation of the meson number. As a suitable initial state for our comparison we implement a homogeneous superposition of isolated single spin flips applied to the ground state $|0\rangle$ of the system for given values of $(h_\parallel, h_\perp)$. This can be formally written as

$$|\psi_i^{(N)}\rangle = \sum_{\{j_1, j_2, \ldots, j_N\}} \sigma_{j_1}^x \sigma_{j_2}^x \ldots \sigma_{j_N}^x |0\rangle. \tag{47}$$

$\{j_1, j_2, ..., j_N\}$ thereby describes a ordered tuple of integers taking values between 2 and $L-1$ with differences of neighboring integers $j_n - j_{n-1} > 1$ for $2 \le n \le N$ in order to realize isolated spin flips not located at the boundary. For small values of the transverse field $h_\perp$ this state has large overlap with a N meson configuration. Each meson thereby has the smallest possible extent of a single lattice spacing. The results for the evolution using few kinks exact diagonalization and tensor network methods in a system with open boundary conditions are shown in Supplementary Fig. 5. For comparison we investigate the meson coverage ρM, which is following the convention of the main text closely related to the magnetization in the system. We find very good agreement between both methods for all tested values of $N \in \{1, 2, 3\}$ and small values of $h_\perp = 0.05$. We, moreover, find qualitative features of the evolution like the oscillation frequency of ρM preserved also for larger values of $h_\perp = 0.1$. We can, moreover, identify an additional offset between results of tensor networks respectively to predictions of exact diagonalization though. This deviation is in agreement with our expectations of decreasing accuracy for larger values of $h_\perp$. This indicates that simulations using exact diagonalization in the few kinks subspace can be used to characterize the physics of a system of few mesons in the limit of small transverse field $h_\perp$.

[1] P. Calabrese, F. H. L. Essler, and M. Fagotti, Quantum quench in the transverse-field ising chain, Phys. Rev. Lett. **106**, 227203 (2011).
[2] S. B. Rutkevich, Energy spectrum of bound-spinons in the quantum ising spin-chain ferromagnet, Journal of Statistical Physics

131, 917 (2008).

[3] S. Scopa, P. Calabrese, and A. Bastianello, Entanglement dynamics in confining spin chains, Phys. Rev. B **105**, 125413 (2022).

[4] M. Kormos, M. Collura, G. Takács, and P. Calabrese, Real-time confinement following a quantum quench to a non-integrable model, Nature Physics **13**, 246 (2017).

[5] A. Bastianello and P. Calabrese, Spreading of entanglement and correlations after a quench with intertwined quasiparticles, SciPost Phys. **5**, 33 (2018).

[6] J. Kogut and L. Susskind, Hamiltonian formulation of wilson's lattice gauge theories, Phys. Rev. D **11**, 395 (1975).

[7] F. M. Surace, P. P. Mazza, G. Giudici, A. Lerose, A. Gambassi, and M. Dalmonte, Lattice gauge theories and string dynamics in rydberg atom quantum simulators, Phys. Rev. X **10**, 021041 (2020).

[8] P. Fendley, K. Sengupta, and S. Sachdev, Competing density-wave orders in a one-dimensional hard-boson model, Phys. Rev. B **69**, 075106 (2004).
